# Are the Editors faced with e-problems performing their duties and responsibilities satisfactorily?

**DOI:** 10.12669/pjms.295.4179

**Published:** 2013

**Authors:** Shaukat Ali Jawaid, Masood Jawaid

**Affiliations:** 1 Shaukat Ali Jawaid, Managing Editor, Pakistan Journal of Medical Sciences, Karachi - Pakistan.; 2 Dr. Masood Jawaid, MCPS, MRCS, FCPS, Assistant Professor Surgery, Department of Surgery, Dow University of Health Sciences, Karachi – Pakistan.

**Keywords:** Electronic publishing, Manuscript Management Software’s, e-problems

## Abstract

Astonishing revolution in information technology, developments in electronic publishing and availability of manuscript management software’s has provided lot of facilities to authors, reviewers as well as editors but it has also given birth to lot of e-problems. This communication highlights some of these e-problems besides discussing the manuscript management system practiced by Pakistan Journal of Medical Sciences through modified Open Journal System. It also cautions the editors of small journals faced with financial and human resource constraints to keep themselves abreast of all these developments, go for automation in e publishing gradually as all the stake holders i.e. authors, reviewers and office management staff learns these and become used to it.

 Publishing and editing a good quality peer reviewed medical journal is quite frustrating and stressful job in developing Third world countries faced with financial as well as human resources constraints which has now been very well documented.^1^^-^^5^ Now with the advent of e-publishing in electronic era many software’s are available which offers numerous advantages but it has also created lot many problems for the editors who remain over-worked with too many submissions and a large number of approved manuscripts waiting for publication. Ahmad Badar has highlighted some of these problems i.e. lack of emotions in this system, switch over from “pen and paper editors” to “electronic editors” and tremendous increase in workload of the Editors.^6^ The electronic publishing offers lot of comfort not only to the editors who can work twenty four hours a day, seven days a week sitting anywhere but also to the authors who do not take days to write or respond to reviewers comments but respond immediately and reviewers who review the manuscripts and return manuscripts with track changes in word document within few days. This increased workload on the editors is likely to affect the quality of the manuscripts accepted for publication and eventually standard of the journal which are faced with financial constraints and limitaions of human resources.

 Some of the complaints which the authors have against editors include no acknowledgement of receipt of manuscripts and no communication as to the further processing of their manuscripts. While the new software’s which is now being increasingly used by many journals (not 100% as yet) has overcome some of these problems as the authors get automatic acknowledgement and they can check the state of their manuscript themselves directly visiting the journal website with the password which they get, they remain impatient and would like to see their papers in print without realizing and being aware of the whole process. We modified the Open Journal System to suite our requirements so that one can manage everything with the few staff members which we have about five years ago and have been gradually going for automation in every step of publishing. This has not been an easy task. The objective of this communication is to share some of the problems which we have faced and how we have tried to solve them for the benefit of colleagues who may be facing similar problems.

**e-Journal System:** ^7^ The modified Open Journal System^8^ which we are using offers full automation but we are trying to adopt this gradually as we, our authors and reviewers learn to use this system. To begin with we used to ask for exclusive online submissions but would also accept submissions through e mails and then upload these ourselves. It went on like this for about six months when we asked the authors to go for direct submissions on our website. Some of the authors are still not used to it and they have to be guided which at times is again a frustrating experience since we as Editors cannot teach them how to use computers and these software’s.

**Editor’s Triage and initial screening:** Ever since we have gone online, there has been enormous increase in submissions which is increasing every year.^9^ We have our own limitations as regards financial and human resources to process these manuscripts. Hence, during initial screening, we do not accept about 70% of the submitted manuscripts for further processing which means they are rejected at these initial stages. The idea is not to keep the authors waiting so that they can submit their manuscripts to some other journal without wasting any further time. As per policy of the journal, we do not accept KAP studies, survey reports, animal studies. We are very selective in accepting Case Reports and Special Communications and Reviews, Meta Analysis which are quite interesting and cover some important topics which we feel will be of interest to our readers are accepted for further processing. Those authors whose manuscripts are not accepted for further processing are politely thanked for taking interest in Pakistan Journal of Medical Sciences and they are told that their manuscript will have a very low priority with us and we do not want to keep them waiting, or in case of overseas manuscripts, we feel it is more suitable for local publication, hence we are sorry to disappoint them. We do not comment on the quality of these manuscripts at all.

**Screening for plagiarism:** As a policy we accept upto three thousand (3000) words for original manuscripts, fifteen hundred words for Case Reports and Special Communications and upto three thousand five hundred words (3500 words) for Reviews, sometimes however there are exceptions. We use different software’s to check plagiarism. Some of them are available free on the net though they are not so good. Some of our reviewers particularly those overseas and those who are affiliated with teaching institutions do have the facility of checking for plagiarism and they do inform us. All such manuscripts which have similarity index which is not in acceptable limits are rejected. Our experience shows that any manuscript which is too lengthy or those with over three thousand words does contain some plagiarism. After signing an agreement with CrossRef for DOI numbers, we have also signed an agreement with CrossCheck for use of their software to check plagiarism. We have now started screening the manuscripts for plagiarism before they are sent for external review with this newly acquired software. All this has also added to the cost of production but it does offer the advantage of ensuring that no plagiarized manuscript gets published.

**Formatting of Manuscripts:** We usually check the submissions on the e journal system after every couple of days (not daily) or may be after a week or ten days if the backlog has not yet been cleared. All the manuscripts which are accepted for further processing after initial screening are then formatted and it does take some time. But the authors are very impatient and when they see their manuscripts are still awaiting assignment on the website, they start sending e mails why they are not being processed further. Hence responding to these e mails adds to the workload. After formatting, these manuscripts are then uploaded on the system again and now they are ready to be sent for external review. Even at this stage some of these manuscripts need some editing and corrections but the authors do not know about this additional work which the editorial team has to perform.

**External Peer Review:** We have over two hundred fifty people on our reviewer’s data base within the country and overseas. Some of them, particularly overseas reviewers are quite expert in doing online reviews, hence they are sent the manuscripts through e journal system but others are sent through e mails with attachments along with the reviewers Performa. Among the reviewers in Pakistan about 20% would still like to receive the print copies for review and we have no option but to oblige them as they all do this honorary. It is not uncommon that one has to send repeated reminders to the reviewers as a vast majority of them won’t respond within the given time. Some of them particularly reviewers in Pakistan at times even misplace the manuscripts and they have to be sent manuscripts again to some by e mail and to others printed copy of the manuscripts. Our efforts continue to increase the Reviewers data base and include those reviewers who can do electronic review and hope to switchover to complete electronic reviewing in the next six months. Till then we have to use both these systems. Our experience with young faculty members has been very good, they are keen, enthusiastic and do a good job in time. Some authors who have published a few manuscripts and their manuscripts are rated of good quality are also invited to join the reviewers data base and we have found it a good source of inducting new reviewers from Pakistan as well as overseas. In some cases, we also give the option to the authors to suggest atleast two reviewers and sometimes do utilize their services particularly in those specialties in which we do not have many or a few reviewers.

**Fast track processing facility:** At times the authors are really faced with an emergency situation like the postgraduates who may have to sit in the fellowship examination and needs published paper to be eligible or someone has to appear in an interview for faculty position and needs published manuscripts. In some other cases someone may need additional published papers to become eligible for further faculty promotion. As the peer review takes atleast four months time and despite best efforts we have not yet been able to reduce this processing time, we have offered the facility of fast track processing for those authors who need early publication. They have to pay extra processing fee but it does not guarantee acceptance or early publication which is made clear to them. They are promised to get their manuscripts peer reviewed within eight weeks time and the reviewers comments are conveyed to them within this time. It depends on the quality of their manuscript; it may be accepted, rejected or asked to revise it. In principle, we do not encourage authors to opt for fast track processing unless it is extremely essential. We have also noted that sometimes some authors have a tendency to misuse this facility. Despite the fact that they have no such urgency, just to get their manuscripts processed speedily, they insist on fast track processing. If such requests are accepted it will deprive many other authors who cannot afford to pay extra to get their manuscripts processed and published quickly. It is a very difficult decision at times and one has to be extremely careful as it should not give an impression that the journal is most interested in financial gains only.

**Reviewing the Reviewers:** Editors are not supposed to work as “Post Office” and as stated by Ahmad Badar “An editor is the reviewer-in-chief, chief justice, solution finder, mentor and guide”,^6^ Hence the editor has to perform these multiple jobs and also try to keep every one, authors, reviewers and readers happy, not an easy job. One of the important task of the Editor related to manuscript management is reviewing the reviewers. Some of the reviewers are excellent, do an exceptionally good job guiding the authors how to improve their manuscripts. Using track changes, some of them even edit, correct and suggest changes which the authors can just accept through the click of a button if they agree. Some of the reviewers fill in the Reviewers Performa which is quite helpful and offers ease in reviewing but there are others who will communicate their comments through e-mails sending their comprehensive reviews through attachments. We do not forward these reviewers’ comments as such to the authors but review their reviews. While their comments are saved as such but those forwarded to the authors are the edited version because at times some of the harsh comments have to be toned down, some of the comments are meant for the editor and not authors. All this takes lot of time. For the authors the manuscript management software’s are quite helpful and in some cases, they make the changes, corrections, additions and return the manuscript within hours and in some cases in the next two three days and then all this gets piled up for the Editorial team to look at it further. However, the authors are again very impatient and wish that the Editor convey them the decision of acceptance as quickly as they have responded which is not possible. In case the reviewers have made track changes and made some other comments and suggestions on the manuscript, the authors are asked to make all the changes in the same file which is being sent to them and highlight all changes or additions made. Not only that they are also asked to convey through a separate communication how they have responded to these comments point wise. Once the revised manuscripts are received, they are looked at by the editorial team and the Editor himself. If the response is satisfactory, an acceptance e mail is sent to the author immediately in some cases within hours or the next day. Some of these manuscripts have to be revised numerous times( in some cases half a dozen times) before they are finally accepted for publication and it may take from twelve to eighteen months from the time of submission to final publication. There is no straight forward Acceptance or Rejection. If the manuscripts are finally rejected after peer review, they are given reasons for rejection. We practice open peer review system for the last seven years and the reviewers comments are conveyed to the authors along with the name of those who have reviewed it. Since a vast majority of our reviewers are from overseas, we never have had any problem. Only in a few cases, the reviewers from Pakistan wished that their identity should not be disclosed and it was accepted. In some of the disciplines we do find difficulty in finding good reviewers and in such cases, we ask the authors to suggest at least two reviewers and it works. The final authority to accept or reject the manuscript lies with the Editor who is eventually responsible for the quality of contents, hence these reviewers are reviewed carefully before taking any decision. Of course the Editor can take a decision contrary to the advice of the Reviewers as their comments are an advice and suggestion as regards acceptance or rejection of a manuscript. It has happened in a very few selected cases with us. However, it must be added here that a journal is only as good as its Editorial Team and Reviewers who remain an asset for the journal. The Editor also has a role in resolving disputes if any between reviewers and authors. The authors can also appeal to the Journal Ombudsman against the decision of the Editor or redress of their grievances’ if any against the journal.

**Reward for the Reviewers:** We do not have enough resources to offer any honorarium to the reviewers or reward them financially. However, we do offer 50% discount to our reviewers in publication charges if they submit their manuscripts for publication. They are also offered 50% discount in fast track processing or at times the processing fee is waived off. In some cases some of their manuscripts may be published complimentary. In every issue, we do have quite a few such manuscripts wherein the authors have been offered some discount or waivers and decision is taken on case to case basis depending on the financial viability. We try to keep contact with our reviewers, meeting some of them within the country personally thanking them for their patronage, help and assistance in this academic activity and those overseas are sent e mails expressing our thanks. This we feel is extremely important to maintain some emotional touch instead of leaving everything to automation offered by these manuscript management software’s which certainly lack emotions. In some exceptional cases some of the reviewers have also been invited to join the Editorial Board which is a very small token of appreciation and recognition of their work.

**Improvement of English language and Grammar:** About 25-30% of the manuscripts which are accepted for further processing does need improvement of English language and Grammar as well. As we follow an author friendly policy, we do request our reviewers and sometimes our editorial team will do this job. The idea is not to discourage people but encourage the authors as far as possible and if they can be helped, we try to do that. However, in some cases the manuscripts need extensive revision and rewriting for which the editorial team certainly has no time; they are returned to the authors with suggestions.

 About 75% of the manuscripts accepted for further processing and external review (out of 30% of the overall new submissions) do get accepted after minor changes and corrections which are done by the Reviewers or by the editorial team but another 15% are accepted after one or more than one revisions. The remaining 10% eventually get rejected after peer review and even revision. This is not liked by authors and it is not uncommon to receive some uncharitable comments and even at times threatening e mails and the poor Editor has to live with all this. 

**Final Editing:** Once the revised manuscripts or those recommended for acceptance by the reviewers are received, they go through the final editing process. Even at this stage, one may come across serious flaws and problems. As the quality of reviewers varies some being excellent, some good and some not so good, the overall responsibility for the quality of manuscripts accepted for publication lies with the Editor, hence one has to be extremely careful. During editing, one may come across that the reviewer did not check some of the figures carefully, there is difference in total in tables, and some of the references marked in the text do not tally with the references in the list at the end. Many of the small journals do not have the luxury of having separate copy editors and even this job has to be performed by the editor. Hence one has to be cautious, check the tables, figures, references carefully. On some occasions we did detect serious problems with the references at this stage and the manuscripts are returned to the authors to recheck all the references once again even if there is problem with one or two references. If one reference is marked wrongly, there is a possibility that all the reference numbers may have to be changed. The bibliographer is also asked to re-check all the references in most of the accepted manuscripts to eliminate any chances of mistake.

**PDF files for Proof Reading:** Once a manuscript goes through all those processed, the final edited copy is passed on for composing and page make-up. The pdf file is then sent to the authors with e-journal system to convey the corrections if any. Here again some of the authors do not know how to convey the corrections. Some are not familiar with how to do corrections in the pdf file. Some do that, others send the corrections by e mail but surprisingly a few will send the original manuscript again after making the changes. It is very frustrating and the authors are informed that we cannot go on formatting their manuscript again and again. They have to convey the corrections so that those changes can be made in the database, sometimes one has to struggle with a few authors to make them understand how and what they are supposed to do and there is no end to some of these e-problems which the editor has to face.

**e-publication ahead of Print:** Once about six to eight manuscripts of an issue are finalized after correction, we publish them online on our website ahead of print. This facility has been welcomed by the authors as they can take a print, give reference since only page number is missing while it has the Volume, number and the year is also mentioned apart from DOI number. Giving references of such manuscripts ahead of print is now a well accepted reality. Then as we continue to receive the corrections, every manuscript which is finalized, is published straight away at times within an hour of the receipt of corrections to the delight of the authors. This process goes on and once the number of manuscripts which is decided to be included in a particular issue is complete, then we do pagination, make these additions and corrections and then generate the pdf files once again with page number before they are uploaded on the website. 

**Last minute corrections:** The complete issue is uploaded and it offers yet another opportunity to the authors to look at their published manuscript and make sure that all the corrections, changes they had suggested have been made and if not, immediately communicate to the editor so that the left over corrections if any can be made. After about two to three weeks time, the final prints are taken and the issue goes for print. Once the issue has been printed, no corrections are possible in the print issue though some corrections can be made in the online edition on the website at any time. In case of corrections or additions at this stage, the pdf file which is uploaded again carries a note that this was uploaded again on such and such date at the end of the manuscript.

While the editorial team is busy making arrangements for printing of the issue after it is finalized and is published on the website, the authors start sending e mails as to when the print copy will be available. These e mails come not only from the correspondence author who is supposed to communicate with the editor but all the co-authors as well. Similarly while conveying the corrections, at times all the author start sending e-mails separately despite the fact that it has been made clear that only the correspondence author should communicate and reply to all these e-mails which further frustrates the over worked editors and editorial teams. Sometimes the authors change their e mail and fail to communicate this change to the editor with the result that reviewer’s comments or pdf files sent for proof reading have to be re-sent on their new e mail address and all this increases the workload manifold.

**Print copy:** One printed copy is sent to the correspondence author soon after printing but sometimes the address given by the authors is incomplete, hence the copy does not reach them. The authors are reminded time and again that they should give complete postal address but even then the journal is blamed for the failure of the authors who ask for additional copy on a new address and sometime one has no other option but to oblige and in the present era of economic constraints, postal charges are quite exorbitant and the journal has to bear all this.

**XML files for PubMed Central:** Once the issue has been published, we send word and pdf files of the issue to an overseas agency to generate XML files for PubMed Central which has approved Pakistan Journal of Medical Sciences for coverage. As per our agreement starting from January 2013 issue the journal will be covered in PubMed Central and we have submitted XML files of all the issues published in 2013 which will appear on PubMed shortly. All this involves additional cost but offers much increased readership for the authors and greater visibility of the journal which will ensure increased citations which will help improve our Impact Factor.

 The above is a brief summary of the manuscript management system which we follow. While the electronic age and the era of e-publishing has brought with it many advantages and facilities, it has definitely increased the workload of the already overworked editors. All the editors may not be performing their duties and responsibilities to the entire satisfaction of the authors and reviewers but one has to look at the whole working set up, environment and in view of the financial as well as human resource constraints, the Editor faced with this plethora of e-problems, do need some sympathy and encouragement and not the uncharitable remarks and comments from the authors.

**Final word of caution:** For Editors of small journals with meager financial and human resources, too much reliance on electronic management system could prove to be a disaster. Try to keep yourself abreast of all the latest developments and advances in technology but do not take hasty steps in adopting them. Adopt them gradually one by one as your resources permit. Hence, it is advisable to follow author friendly, reviewer friendly policy, do not be so strict with deadlines while dealing with reviewers, gradually switch over to automation as everyone involved i.e the authors, reviewers; office management staff gets trained and used to the system. Slow and steady is most often sure to win the race and the same is true in this publication game.
